# A Beta-mixture model for dimensionality reduction, sample classification and analysis

**DOI:** 10.1186/1471-2105-12-215

**Published:** 2011-05-27

**Authors:** Kirsti Laurila, Bodil Oster, Claus L Andersen, Philippe Lamy, Torben Orntoft, Olli Yli-Harja, Carsten Wiuf

**Affiliations:** 1 Department of Signal Processing, Tampere University of Technology, P.O. Box 527, FI-33101 Tampere, Finland; 2 Bioinformatics Research Centre, Aarhus University, C.F. Møllers Allé 8, DK-8000 Århus C, Denmark; 3 Department for Molecular Medicine, Aarhus University Hospital/Skejby, Brendstrupgårdsvej 100, DK-8200 Århus N, Denmark

## Abstract

**Background:**

Patterns of genome-wide methylation vary between tissue types. For example, cancer tissue shows markedly different patterns from those of normal tissue. In this paper we propose a beta-mixture model to describe genome-wide methylation patterns based on probe data from methylation microarrays. The model takes dependencies between neighbour probe pairs into account and assumes three broad categories of methylation, low, medium and high. The model is described by 37 parameters, which reduces the dimensionality of a typical methylation microarray significantly. We used methylation microarray data from 42 colon cancer samples to assess the model.

**Results:**

Based on data from colon cancer samples we show that our model captures genome-wide characteristics of methylation patterns. We estimate the parameters of the model and show that they vary between different tissue types. Further, for each methylation probe the posterior probability of a methylation state (low, medium or high) is calculated and the probability that the state is correctly predicted is assessed. We demonstrate that the model can be applied to classify cancer tissue types accurately and that the model provides accessible and easily interpretable data summaries.

**Conclusions:**

We have developed a beta-mixture model for methylation microarray data. The model substantially reduces the dimensionality of the data. It can be used for further analysis, such as sample classification or to detect changes in methylation status between different samples and tissues.

## Background

Interest in understanding the effects of epigenetics in relation to different complex diseases is increasing. One epigenetic mechanism of particular interest is DNA methylation at cytosines in CpG dinucleotides. The methylation patterns of genes may change and these alterations have been shown to be related to complex diseases, such as heart diseases [1], schizophrenia [2] and different cancers [3,4]. In cancer, several methylation changes are detectable at the early stages of cancer or even in pre-cancerous tissues or blood [5,6]. In addition, other methylation alterations have been shown to be specific to cancer type and stage [7,8].

High-throughput technologies, such as microarrays and large-scale sequencing, allow genome-wide methylation measurements. Analysis of methylation data requires efficient statistical methods to be able to identify potential methylation biomarkers and differential methylation patterns across sample types. Several methods have been proposed to pinpoint significant methylation differences in patients with cancer and to classify different tissue types. Examples include feature selection methods [9], mixed effect and generalized least square methods [10] and singular value decomposition-based methods [11]. In addition, methylation patterns of distinct microarray probes have also been modeled with beta-mixture models, which subsequently are used with partitioning algorithms to separate different tissue types into clusters [12]. Each probe is modelled with its own beta-distribution that depends on the tissue type only. In the present paper, we propose a beta-mixture model to describe genome-wide methylation. We assume that methylation levels of nearby probes are dependent, as demonstrated previously by [13] and [14], and that methylation can be categorized into three different broad categories or states, low, medium and high methylation. In addition, we include knowledge about the genomic background (proximity to CpG-islands) of the probes following suggestions in [15]. In total, our model has 37 parameters (see Section Data Analysis) per sample compared with approximately 27k probes in Illumina methylation arrays. The parameters comprise genomic background, methylation state and level, and dependency between probes. In short, the model facilitates

(i) reduction of the dimensionality of a methylation profile

(ii) for each probe, computation of the posterior probability of a methylation state,

(iii) computation of a posterior probability that the latter state was correctly predicted.

We apply the model to a set of methylation microarray measurements from colon cancer samples and show that the model parameters reflect global patterns in the data. Based on the estimated parameters, we are able to classify the samples with high accuracy and to exhibit global differences between the cancer samples. Furthermore, the model assigns a methylation state to each probe value. Using the states, accessible data summaries are provided.

## Results and Discussion

### The Methylation Array and the Number of CpGs in Probes in Different Genomic Regions

**Figure 1:**
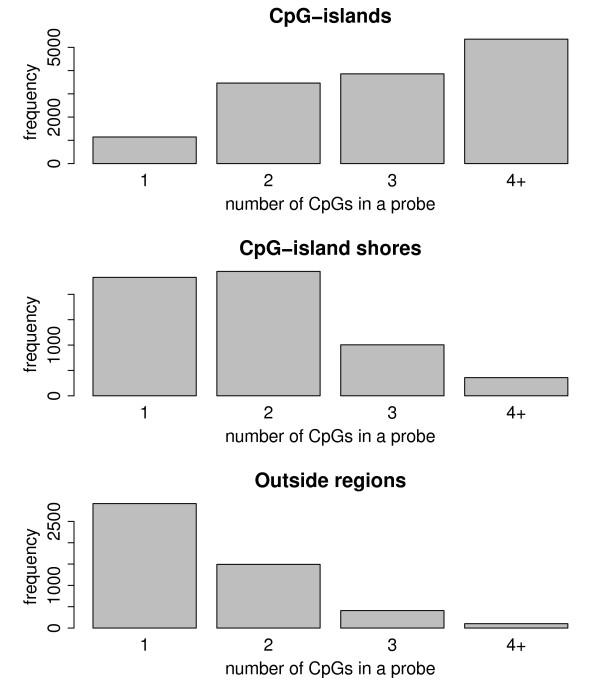
**Frequencies of CpGs**. Frequencies of the number of CpGs in probes according to the location in the genome.

The Illumina human methylation 27k array consists of 27,578 probes that measure the methylation status of CpGs in the human genome at single nucleotide resolution. The array measures genome-wide methylation and the probes target over 14,000 genes. The great majority of the genes included in the array have two methylation probes (80.9%), while 17.6% of the genes have one methylation probe and only 1.5% have more than two probes. The degree of a methylation of each probe is measured by the beta-value which is a continuous variable varying between zero and one, where one means full methylation. The probes of the Illumina human methylation 27k array are 50 nucleotides long and contain different numbers of CpGs. Probe locations can be divided into CpG islands (I), CpG island shore regions (within 2000 bp from CpG-islands) (S) and outside regions (O) (these definitions are adapted from [15]). About one quarter of the probes (6,150) are located in the shore regions, one sixth (4,922) in outside regions and the rest (13,819) in the CpG-islands. Depending on the region, methylation appears to happen at different rates such that CpG-islands are usually less methylated than the CpGs in other genomic regions [15]. Logically, this results in an uneven distribution of methylation in regions with different numbers of CpGs because more CpGs occur in CpG-islands than in outside regions; see Figure 1. As a consequence, the number of CpGs affects the amount of methylation. In fact, we found a negative correlation between the number of CpGs in a probe and the beta-value of the probe (Pearson's correlation coefficient *r *= -0.28). However, this correlation is reduced markedly when studying the probes in I, S or O regions separately (correlation coefficients *r *= -0.049, *r *= -0.053 and *r *= 0.083, respectively). The probe distance from the CpG-island in the O class showed no correlation with the strength of methylation.

### Parameter Estimates and Interpretation

We used the proposed method to analyze methylation microarray data from normal and colon cancer tumor samples. Colon cancer can be divided into different types and here we study patients with microsatellite instable (MSI) and microsatellite stable (MSS) tumors. In addition, we have samples of benign colon adenomas that are not considered as cancer tumors but are classified as MSS-type adenomas. Our data set contains 42 Illumina methylation 27k microarray samples, divided into 6 normal, 6 adenoma, 6 MSI and 24 MSS-samples.

The basic idea behind the model is the assumption that the methylation level of a CpG (probe) can be divided into three different states, low (L), medium (M) and high (H) methylation. The three states are biologically motivated in the following sense. L corresponds to the situation, where (almost) all cells in a sample are unmethylated and H to the situation, where (almost) all cells are methylated, irrespectively of the composition of the cells in the sample. M captures the situation in which the cells are only partly methylated (e.g. hemi-methylation), or some cell types in the sample are methylated while others are not.

**Figure 2:**
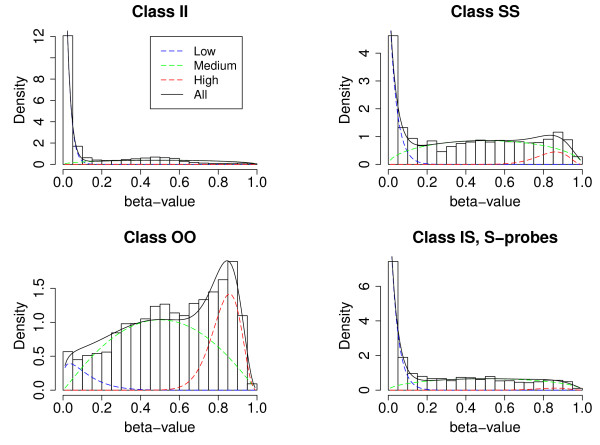
**Beta distributions mixtures**. Mixtures of beta distributions for different probe-type classes for one MSI-sample.

The latter could be the case if the sample consists of different cell types (sample purity) or one cell type shows heterogeneity, as would be expected for cancer cells. The three states are further empirically motivated (Figure 2).

We assume that statistical properties of these different states are the same throughout the genome and that methylation of a CpG site depends on its location (I, S or O) in the genome in relation to the nearest CpG-island. We concentrate on modelling genes with two probes; however, our model can also be extended to genes with more than two methylation probes (for the genes with more than two probes the two first probes were used). The beta-values of a gene's probe pairs are dependent with a correlation coefficient of r = 0.668 between the probes. A similar degree of correlation has been reported for other data sets, while the probes measuring methylation levels of different genes have shown no dependency [16]. We built a model that can be considered a hidden Markov model (HMM) of probe pairs within a gene. The hidden states are the methylation levels, L, M and H. Further, the CpG probe pairs are classified by their locations into classes (I,I), (S,S), (O,O) and (I,S). The latter includes both (I,S) and (S,I) pairs. Other cases were omitted as they contained only a few or no probe pairs. We built one model for each of the classes.

We fitted a mixture of three beta-distributions (distributions corresponding to the low, medium and high methylation states, respectively) for each sample in the colon cancer data set, such that the beta-distribution corresponding to the medium methylation state is symmetrical; as described in greater detail in Methods. The beta-distribution gives the density of the beta-value given the hidden state. That is, for each sample we estimate beta-distribution parameters *α *and *β*, mixture proportions *ω *and a transition probability matrix *T *for the HMM (see Methods for further details). The mixture proportions are the *a priori *probabilities that a probe is found in a given hidden state and the transition matrix gives the probabilities that a probe in some hidden state *k*_1 _= L, M, or H, is followed by a probe in state *k*_2 _= L, M, or H. For the class (I,I) we set the mixture coefficient of the high methylation state to 0, i.e. this state could not be reached as high methylation appears to be very rare in this class. The same is assumed for the I probes in the class (I,S). Figure 2 shows the empirical and the fitted mixture distributions for one MSI-sample for different classes. We also built a model with only two states for every class and a model where the high methylation state was included in all classes but these did not reflect the data properties equally well as the model presented.

**Figure 3:**
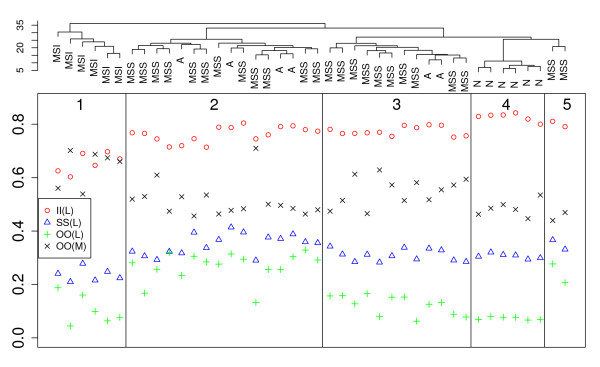
**Hierarchial clustering and mixture proportions**. Top part: Hierarchial clustering of samples using methylation array data (all probes). Lower part: Four mixture proportions . from different classes plotted in the same order as in the cluster dendrogram. N = normal, A = adenoma. L = low methylation state, M = medium methylation state.

**Figure 4:**
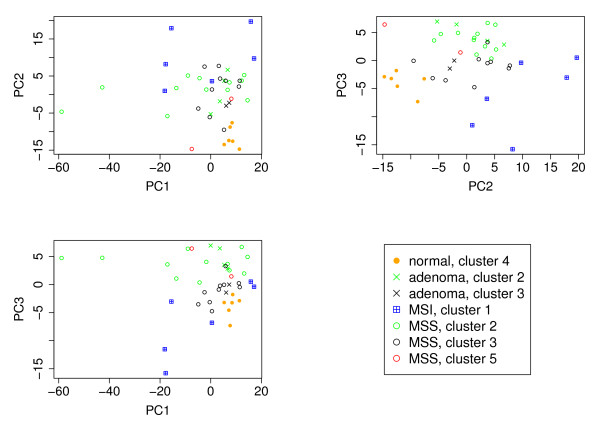
**Principal component analysis on parameters of beta distributions**. Samples are plotted according to the three first principal components (PCs). The clusters are the same as in Figure 3 and the samples belonging to each cluster are plotted with the same color, while the different sample types (normal, adenoma, MSI and MSS) are marked with a similar mark.

The mixture model parameters in different classes varied between samples, but some general trends in the sample groups (normal, adenoma, MSI, MSS) could be seen; see Figure 3 for examples. In the figure, the hierarchial clustering of the samples based on the methylation array data (all probes) is also shown. The adenomas and the MSS cancers are mixed in two big clusters and the differences between these clusters can be seen in the parameters as well, *cf*. Figure 3 and 4.

Regarding the mixture proportions, the group of MSI cancers is most easily discriminated from the other samples (some examples are shown in Figure 3). Indeed, for the (I,I) probe pair class, the MSI mixture proportions for the low methylation state are clearly lower and the mixture proportions of the medium methylation state are higher than in the other classes. Similarly, but less clearly, differences for the shore (S) region probe pairs could be detected, e.g. medium and high methylation state mixture proportions are higher for MSI cancers compared with the other samples (results not shown). Furthermore, many mixture proportions vary in the same range when studying normals, adenomas and MSS cancers. However, normals have slightly higher proportions for low methylation in (I,I) class than the other samples.

The probes in the outside regions show the biggest variations between the groups. Normals have small coefficients for low methylation whereas the high and medium methylation states are almost equally probable. On the contrary, for all tumor samples medium methylation is clearly the most evident and the proportions of the low and high methylation vary greatly. In addition, for some adenoma and MSS cancer samples, low methylation was more probable than high methylation while in MSI cancer samples the low methylation state always had the smallest coefficient. Mixture proportions in the outside regions also distinguished the two clusters of adenomas and MSS cancers well. Overall, MSS cancers and adenomas share similar proportions in all the classes. Class (I,S) mixture proportions do not show as large differences between groups as other classes.

We can see similar differences between groups in the estimated parameters of the beta-distribution as in the mixture proportions. We performed a principal component analysis (PCA) on the vector of beta-distribution parameters (there are 13 for each sample) to illustrate how the parameters vary across samples and tissues. In Figure 4 all samples are plotted based on the three first principal components. Again, normals and MSI cancers are clustered into distinct groups while adenomas and MSS cancers overlap. However, the two clusters of the hierarchial clustering (Figure 3) encompassing adenomas and MSS cancers could be distinguished by the principal components. To further illustrate the use of the model we classified the samples using the mixture proportions and a leave-one-out procedure (Equation 1 in Section Data Sets). We used as classes the four biggest clusters that can be seen in Figure 3 (clusters 1-4). These were obtained by hierarchial clustering. The fifth cluster was omitted as it contained only two samples i.e., altogether 40 samples of the clusters 1-4 were used for the classification analysis. Overall, 32 out of the remaining 40 samples (80%) were classified correctly, see Table 1. For comparison, we followed the same procedure using beta-values from 100, 500, 1000, 2000, and 5000 probes and found similar classification results though with lower performance (see Additional file 1 Tables S6-S10). Using k-means only results generally in better performance: 82.5% using mixture proportions and 90% when using 5000 probes; for fewer probes the performance was less than when using the mixture proportions (see Additional file 1 Tables S1-S6 and Table S11). Note that k-means find the best division of the samples into four clusters, whereas the leave-one-out method assumes clusters are defined and classifies the samples one at a time.

**Table 1:** Sample Classification

True cluster	Total	Predicted as			
		
		1	2	3	4
1	6	5	1	0	0

2	16	1	14	1	0

3	12	0	4	8	0

4	6	0	0	1	5

Classification based on leave-one-out cross validation.

### Data Summaries

In this section we further illustrate the use of the model. For each probe pair, we computed the posterior mixture proportions and the most likely state. The most frequent state in a group was defined as the group's overall state. In Table 2, a summary of the results is shown. There are clear differences between groups which we expect from the previous analysis. Normal samples have almost twice as many HH probe pairs as the other groups. Likewise, MSI cancers have fewer probe pairs with low methylation states, while the medium methylation state is far more common than in the other groups. The distributions of methylation states for adenomas and MSS cancers resemble each other.

**Table 2:** Numbers of probe pairs.

	LL	LM	LH	ML	MM	MH	HL	HM	HH
normal	4934	313	11	1075	1805	247	30	236	860

(%)	51.9	3.3	0.1	11.3	19.0	2.6	0.3	2.5	9.0

adenoma	5121	428	51	964	2066	247	91	203	475

(%)	53.1	4.4	0.5	10.0	21.4	2.6	0.9	2.1	4.9

MSI	3970	382	21	989	3053	273	79	235	541

(%)	41.6	4.0	0.2	10.4	32.0	2.9	0.8	2.5	5.7

MSS	4945	430	32	1004	2290	218	71	188	531

(%)	50.9	4.4	0.3	10.4	23.6	2.2	0.7	1.9	5.5

Average number of probe pairs in different methylation states according to group.

We use the posterior mixture proportions to calculate the false annotation rate, FAR (see Section Data Analysis). FAR measures how often a probe pair is assigned to the wrong state: To each probe pair, we assign the hidden state (*k*_1_, *k*_2_), with *k*_1_, *k*_2 _= L, M, or H, with the highest posterior proportion. The probability that this assignment is incorrect is given by FAR (see Section Data Analysis). For the colon data set, FAR = 0.128, implying that about 1 in 8 probe pairs should have a wrong annotation. For example, in Figure 2, the bottom left plot, wrong annotations are likely to occur when the probability of the low and the medium states are similar (e.g. beta-value around 0.1), while confident annotations are made when e.g. the beta-value is around 0.5.

Next, we selected the probe pairs where the overall group state differed between groups. We further reduced the number of probe pairs by only choosing those for which the posterior mixture proportions showed a significant difference between groups using Fischer's linear discriminant analysis. Table 3 shows the results. For each probe pair, one or both probes might differ between two groups; in the table we count how many probes show a given change, e.g. L→M. Most differences were found between normals and MSI cancers with almost 2000 differences while only 35 changed probes are detected between adenoma and MSS samples. This again shows that based on methylation data, adenomas and MSS cancers are difficult to distinguish. In addition to the number of changes, also the type of changes differed between comparisons.

**Table 3:** Methylation state changes.

	Norm vs Aden	Norm vs MSI	Norm vs MSS	Aden vs MSI	Aden vs MSS	MSS vs MSI
L → M	256	1627	613	673	25	42

M → L	173	87	344	7	8	974

M → H	9	24	24	16	2	152

H → M	168	238	694	4	0	131

L → H	0	0	0	0	0	0

H → L	1	0	0	0	0	0

Total	606	1977	1675	700	35	1299

Number of changes in methylation states in group comparisons. Norm = normal, Aden = adenoma.

For example, when comparing normals with MSI cancers, over 80% of changes were from low to medium methylation. In comparison, between normals and adenomas and normals and MSS cancers, the proportions were 42% and 37%, respectively.

The characteristics of the changed probes also differed between comparisons; see Table 4. For all comparisons except between normals and MSS cancers the majority of methylation changes happened at CpG-islands. Further, there are differences between adenomas and MSS cancers which may be used to distinguish between the two tissue types.

**Table 4:** Number of changes in different regions in group comparisons.

	Norm vs Aden	Norm vs MSI	Norm vs MSS	Aden vs MSI	Aden vs MSS	MSS vs MSI
I	272	1356	555	527	27	595

(%)	45.0	68.6	33.1	75.3	77.1	45.8

S	87	377	183	148	6	285

(%)	14.4	19.1	10.9	21.1	17.1	21.9

O	246	244	937	25	2	419

(%)	40.6	12.3	55.9	3.6	5.7	32.3

Total	606	1977	1675	700	35	1299

Norm = normal, Aden = adenoma.

## Conclusions

In this paper, we have proposed a model for microarray methylation data. The model uses four different probe pair classes and three different methylation states. It is motivated by the empirical distribution of beta-values and knowledge of the genomic content of CpG dinucleotides. It reduces the dimensionality of a microarray data set to 37, the number of parameters in the model. The model allows us to assign one of three broad classes (low, medium or high) to each methylation probe value and assess the correctness of the assignment.

Further, we illustrate the use of the model by analysing a colon cancer data set. Normal and MSI samples could easily be distinguished from the other samples, but adenomas and MSS cancers were mixed together. However, the hierarchial clustering based on all beta-values (27k probes) also mixed these two groups. This suggests that the methylation patterns in adenomas and MSS cancers are very similar, which is in agreement with previous studies [17,18]. In addition, we identified differences in the genomic localisation of methylation changes. This observation may be used to discriminate between adenomas and MSS cancers from genome-wide methylation data.

In the future it would be interesting to integrate information from different data sources, such as methylation, gene expression and copy numbers, into one model. It may also be beneficial to take the full step and model at the level of the DNA sequence directly, anticipating the rapidly growing interest in next-generation sequencing.

## Methods

### Data Sets and Preprocessing

We used a data set that consists of 42 Illumina methylation 27k microarray samples, that can be divided into 6 normal, 6 adenoma, 6 MSI and 24 MSS-samples. Raw data was preprocessed and normalized with the background method using Illumina's BeadStudio software. After this the methylation value for each probe (the beta-value) was computed with the formula

where *M *is the value of the methylated bead type probe and *U *is the value of the unmethylated bead type probe. Beta-values vary between zero and one. In the analysis, we omit probes with *β *= 0, these are generally of bad quality.

### Model

Let *s_j _*be the location (I, S or O ) of the *j*th probe in the genome and let *x_ij _*be the beta-value computed for the probe *j *of sample *i*. Based on the locations, the data is divided into classes *C_a _*= {*j*|*s_j _*= *a*}, where *a *∈ {I, S, O}. For each class, we assume that the data follows a beta mixture model:

Here, *k *= L, M, H can be considered hidden (unknown) states and *ω_aki_*, the *a priori *probability that a probe from sample *i *and class *a *is in state *k*. The *ω_aki_*'s are called mixture proportions for sample *i *and class *a*, and fulfill ∑*_k _ω_aki _*= 1. If a probe is in hidden state *k*, it emits a methylation value according to a beta-distribution with parameter (*α_aki_*, *β_aki_*) and normalizing constant *B*(*α_aki_*, *β_aki_*). We assume that the beta-distribution of the medium methylation state is symmetric, i.e. *α*_*a*M*i *_= *β*_*a*M*i*_. Throughout the paper *k *refers to the methylation state, *k *= L, M, H.

**Figure 5:**
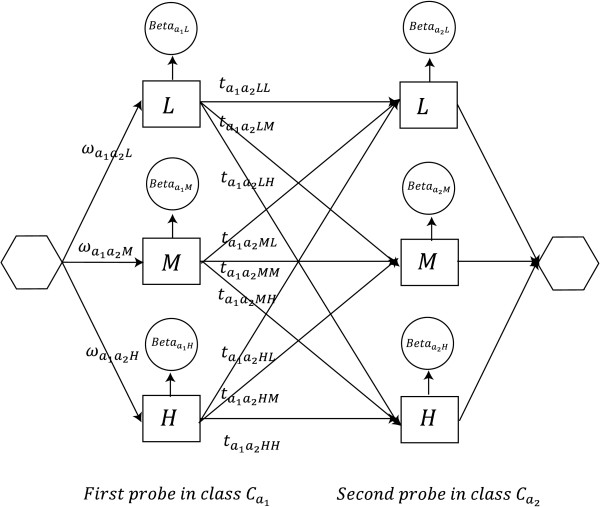
**Graphical view of the model**. The first probe of a probe pair belonging to class (*a*_1_, *a*_2_) reaches the states L, M and H with probabilities ,  and , respectively, and each state emits a methylation value from a corresponding beta-distribution (denoted in the figure by ). Sample index *i *is suppressed for clarity. Then, transition to the state of the second probe happens according to the transition probabilities  and similarly to the first probe, a methylation value is emitted from a beta-distribution.

To take dependencies between neighboring probe into account we do the following (for an illustration, see Figure 5). For a probe pair (*x_ij_*, *x*_*i*(*j*+1)_), i.e. the first two probes in a gene, we model the two probes assuming Markov dependency between them,

Here, (*a*_1_, *a*_2_) denotes the class of the probe pair,  is short for the normalizing constant  of the corresponding beta-distribution, and  is the *a priori *probability (mixture proportion) that the probe pair has (hidden) methylation state (*k*_1_, *k*_2_). There are nine such hidden states.

The bottom line in Equation 1 gives the density of the paired methylation values (*x_ij_*, *x*_*i*(*j*+1)_) as a mixture distribution over the nine hidden states. Given the probe pair is in hidden state (*k*_1_, *k*_2_), the methylation values are emitted independently according to two beta distributions. The parameters of the beta-distribution are assumed to depend only on the corresponding probe, not the probe pair, and again we assume symmetry for the medium methylation state, i.e. , *l *= 1, 2. The middle line shows the same density written as a Hidden Markov Model. The first probe is in state *k*_1 _with probability , while the second probe is in state *k*_2 _with probability  (given the first is in *k*_1_). Thus,  is a 3 × 3 transition matrix for each class (*a*_1_, *a*_2_) and sample *i*.

If Equation 1 is marginalized to obtain the density for a single probe, we find Equation 1 with , where  is the empirical frequency of class (*a*_1_, *a*_2_) among the probe pairs. Similarly for the second probe in the pair.

### Data Analysis

Model parameters are estimated using maximum likelihood. Briefly, first the beta-distribution parameters are defined for each probe class (*C*_I_, *C*_S_, *C*_O_) and for each state (L,M,H) using R's optim-function and EM-algorithm, the parameters are obtained according to Equation 1. Secondly, for each probe pair, the obtained beta-distribution parameters are used to estimate the mixture parameters  and transition probabilities of the matrix *T *. In this step, the Baum-Welch algorithm is used. After the model estimation, parameters are obtained that include 13 beta-distribution paratemers (medium methylation distribution is symmetric, i.e., only one parameter is needed) for L, M and H states, 7 mixture proportion parameters for probe classes (I,I), (S,I), (S,S) and (O,O) (one for the class (I,I) as high methylation cannot be obtained and two for the other classes) and 17 transition probabilities for the four probe pair classes (6 parameters for (S,S) and (O,O), 3 for (S,I) and 2 for (I,I)).

The most likely methylation states for each probe pair are computed with the Viterbi algorithm, in addition, we compute posterior probabilities for each possible state combination for each probe pair. We exclude the classes (S,O), (O,S), (I,O) and (O,I) from the analysis because there are very few probe pairs in these classes (121 in total). For convenience, we group (S,I) and (I,S) together by swapping the probes of the latter. In this way we are left with four classes, (I,I), (S,S), (O,O), and (S,I).

For the classification of the samples using estimated parameters, we apply a leave-one-out method [19]. If there are *G *different groups, then sample i is classified as belonging to the group that minimizes the Mahanalobis distance(1)

where *ν_ti_*s, *t *= (*a*_1_, *a*_2_, *k*_1_, *k*_2_), are the mixture proportions of the different two probe classes, *μ_tg _*and  are the empirical mean and variance of the *ν_ti_*s over all samples *i *in group *g*. If sample *i *belongs to group *g*, then it is left out when calculating the mean and the variance of that particular group. In addition, we performed the same procedure using beta-values from 100, 5000, 1000, 2000, and 5000 probes. Also we used k-means with the same number of beta-values. These were selected as those having the largest variance among all the probes. Note that the first approach assumes we know the groups, while in the second approach k-means finds the optimal division of samples into four groups.

We compute a false annotation rate (FAR) for the data which we define similarly to the false discovery and the false negative rates in [20]; that is, for a set *J *of probe pairs (with cardinality #*J*) the FAR is defined by(2)

where(3)

is the posterior probability of the most probable state of probe pair (*j*, *j *+ 1) with methylation values (*x_j_*, *x*_*j*+1_) and(4)

are the posterior mixture proportions given (*x_j_*, *x*_*j*+1_), calculated with the Viterbi algorithm. The FAR is a natural measure here as it provides the posterior probability (i.e. given the methylation values) that a probe pair is classified as being in hidden state (*k*_1_, *k*_2_), when in fact it is in .

We used Fischer's linear discriminant analysis [21] to test for differences in posterior mixture proportions between groups. To assess the significance of the test statistics we permuted group labels 10 000 times and redid the analysis. We used a significance level of 1%.

Programs used for statistical analysis were written in R http://www.r-project.org/ and are available upon request.

## Authors' contributions

KL did the analysis and drafted the paper. KL and CW designed the study and wrote the paper. BO, CLA, PL and TO generated the data and OY-H contributed discussion. All authors have read and approved the final manuscript.

## Supplementary Material

Additional file 1 **Tables S1-S11**. Classification results using k-means clustering and the leave-one-out method with Mahalanobis distance. In Tables S1-S10, 100-5000 probes with the highest variance across samples were used in the analysis. Table S11 shows the results of k-means classification using the mixture proportions only (as in Table 1). Clusters are the same as in Figure 3 and 3 in Table 1. Click here for file
