# Supplementary material for: A Beta-mixture model for dimensionality reduction, sample classification and analysis
Source: BMC Bioinformatics. 2011 May 27;12:215. doi: 10.1186/1471-2105-12-215 (PMC3126746; doi:10.1186/1471-2105-12-215)
Supplement: Additional file 1 — Tables S1-S11. Classification results using k-means clustering and the leave-one-out method with Mahalanobis distance. In Tables S1-S10, 100-5000 probes with the highest variance across samples were used in the analysis. Table S11 shows the results of k-means classification using the mixture proportions only (as in Table 1). Clusters are the same as in Figure 3 and 3 in Table 1. [file 1471-2105-12-215-S1.PDF]

A Beta-Mixture Model for Dimensionality Reduction, Sample Classification and Analysis

Kirsti Laurila Bodil Oster Claus L Andersen Philippe Lamy
Torben Orntoft Olli Yli-Harja Carsten Wiuf

Additional File 1

Classification results using k-means clustering and the leave-one-out method with Mahalanobis distance (see Data Analysis). 100-5000 probes with the highest variance across samples or the mixture proportions were used in the analysis. Clusters are the same as in Figure 3 and in Table 1.

Supplementary Tables

Table S1

Classification based on k-means clustering with 5000 probes. Performance: 90% correct.

True cluster	Total	Predicted as 1	2	3	4
1	6	6	0	0	0
2	16	2	13	1	0
3	12	0	0	11	1
4	6	0	0	0	6

Table S2

Classification based on k-means clustering with 2000 probes. Performance: 67.5% correct.

True cluster	Total	Predicted as 1	2	3	4
1	6	6	0	0	0
2	16	1	8	7	0
3	12	0	4	7	1
4	6	0	0	0	6

Table S3

Classification based on k-means clustering with 1000 probes. Performance: 65% correct.

True cluster	Total	Predicted as 1	2	3	4
1	6	6	0	0	0
2	16	3	7	6	0
3	12	0	4	7	1
4	6	0	0	0	6

Table S4

Classification based on k-means clustering with 500 probes. Performance: 65% correct.

True cluster	Total	Predicted as 1	2	3	4
1	6	6	0	0	0
2	16	3	7	6	0
3	12	0	4	7	1
4	6	0	0	0	6

Table S5

Classification based on k-means clustering with 100 probes. Performance: 60% correct.

True cluster	Total	Predicted as 1	2	3	4
1	6	6	0	0	0
2	16	4	6	6	0
3	12	0	4	6	2
4	6	0	0	0	6

Table S6

Classification based on the leave-one-out method with Mahanalobis distance with 5000 probes. Performance: 77.5% correct.

True cluster	Total	Predicted as 1	2	3	4
1	6	5	1	0	0
2	16	0	16	0	0
3	12	0	6	6	0
4	6	0	0	2	4

Table S7

Classification based on the leave-one-out method with Mahanalobis distance with 2000 probes. Performance: 75% correct.

True cluster	Total	Predicted as 1	2	3	4
1	6	4	2	0	0
2	16	0	16	0	0
3	12	0	6	6	0
4	6	0	0	2	4

Table S8

Classification based on the leave-one-out method with Mahanalobis distance with 1000 probes. Performance: 67.5% correct.

True cluster	Total	Predicted as 1	2	3	4
1	6	3	3	0	0
2	16	0	14	2	0
3	12	0	6	6	0
4	6	0	0	2	4

Table S9

Classification based on the leave-one-out method with Mahanalobis distance with 500 probes. Performance: 72.5% correct.

True cluster	Total	Predicted as 1	2	3	4
1	6	5	1	0	0
2	16	0	14	2	0
3	12	0	6	6	0
4	6	0	0	2	4

Table S10

Classification based on the leave-one-out method with Mahanalobis distance with 100 probes. Performance: 67.5% correct.

True cluster	Total	Predicted as 1	2	3	4
1	6	5	1	0	0
2	16	0	14	2	0
3	12	0	7	5	0
4	6	0	0	3	3

Table S11

Classification based on k-means clustering with mixture proportions. Performance: 82.5% correct.

True cluster	Total	Predicted as 1	2	3	4
1	6	5	1	0	0
2	16	1	14	1	0
3	12	0	0	8	4
4	6	0	0	0	6
